# Desperate for Sleep: Exploring Parental Perceptions of Melatonin Use Among Adolescents With Neurodevelopmental Disorders

**DOI:** 10.1111/jspn.70010

**Published:** 2025-11-01

**Authors:** Alyson E. Hanish, Shelby M. Freudenburg, Abbey J. Klein, Danielle J. Stappert, Marcia Y. Shade

**Affiliations:** ^1^ College of Nursing University of Nebraska Medical Center Omaha Nebraska USA

**Keywords:** attention‐deficit hyperactivity disorder, autism spectrum disorder, melatonin, neurodevelopmental disorder, sleep

## Abstract

**Purpose:**

Sleep disturbances are common in adolescents diagnosed with neurodevelopmental disorders (NDDs), such as autism spectrum disorder (ASD) and/or attention‐deficit hyperactivity disorder (ADHD). Sleep‐related impairment can include worsening of underlying symptoms such as stereotypic and repetitive behavior, inattention, hyperactivity, and impaired learning. Exogenous melatonin has shown promise in improving sleep onset latency and total sleep time in adolescents with NDDs. The purpose of this study was to describe parents' experiences with sleep disturbance, sleep‐related impairment, and exogenous melatonin use in their adolescent child diagnosed with an NDD.

**Design and Methods:**

We conducted semi‐structured interviews with twenty‐six parents of adolescents (11–18 years of age) diagnosed with ASD and/or ADHD. Data were analyzed using qualitative conventional content analysis.

**Results:**

Before the initiation of melatonin, all parents stated their child had experienced difficulties falling asleep. Parents described sleep‐related impairments in both child and family functioning, such as behavioral issues, cognitive difficulty, and parental lack of sleep. Most parents had attempted sleep hygiene‐related interventions before initiating melatonin. Over half of the parents reported initiating melatonin at their healthcare provider's recommendation. All parents believed melatonin was natural, without major health or safety issues. All parents described subjective improvements in their child's sleep latency with the use of exogenous melatonin. Parents of adolescents diagnosed with ASD and/or ADHD reported that melatonin is a safe and effective intervention for the management of their child's sleep disturbances. Parents also reported switching melatonin brands, changing melatonin dosages, and discontinuing and restarting melatonin.

**Practice Implications:**

Due to the known reported inconsistencies in purity and dosage between over‐the‐counter melatonin brands, frequent switching of brands and dosages should be a consideration for patient education and the potential impact of efficacy and safety. Practice would benefit from future studies targeting validation and standardization of age‐appropriate adolescent sleep‐related intervention, including the everyday usage and long‐term safety of exogenous melatonin.

## What Is Currently Known?

Over the past decade, families, teachers, clinicians, and researchers have reported inadequate sleep in adolescence. Sleep disturbances are more common in children and adolescents diagnosed with neurodevelopmental disorders (NDDs), such as autism spectrum disorder and attention‐deficit hyperactivity disorder, and impact underlying symptoms. The high prevalence of sleep problems in children with NDDs and the potential negative consequences of sleep disturbance make appropriate intervention an urgent priority for many families.

## What Does This Article Add?

In most countries, exogenous melatonin is not available over‐the‐counter, requiring a prescription. Over‐the‐counter availability in the United States may lead to different patterns of exogenous melatonin usage and perceptions among parents. The purpose of this study is to describe parents' perceptions of exogenous melatonin usage in their older children (adolescents 11–18 years of age) with NDDs.

## 1 Introduction

Sleep disturbances are common in children and adolescents diagnosed with neurodevelopmental disorders (NDDs) (e.g., autism spectrum disorder and attention‐deficit hyperactivity disorder), with an estimated prevalence of 50%–80%, which is higher than the general pediatric population (Belli et al. 2022; Blackmer and Feinstein 2016; Shelton and Malow 2021). Sleep disturbances in children and adolescents with NDDs include bedtime resistance, long sleep onset latency, frequent nighttime awakenings, poor sleep efficiency, and less‐than‐optimal total sleep time (Belli et al. 2022; Malow et al. 2016; Shelton and Malow 2021). In addition to the functional impairments associated with sleep deprivation, sleep disturbances in children and adolescents with NDDs may further aggravate underlying NDD symptomology (e.g., repetitive and stereotypic behavior, inattention, and hyperactivity, as well as interfere with learning and cognition) (Cohen et al. 2014; Shelton and Malow 2021). Families may also be negatively impacted by a child's sleep problems, leading to increased maternal stress (Levin and Scher 2016), diminished parental sleep, and impaired family functioning (Jan et al. 2008; Lopez‐Wagner et al. 2008; Meltzer 2008).

Over the past decade, families, teachers, clinicians, and researchers have reported inadequate sleep in adolescents (Healthy People 2030 and U.S. Department of Health and Human Services 2024; Owens et al. 2014; Uccella et al. 2023a). Healthy People 2030 prioritized sleep health as an important area for public health research, addressing the need to increase the proportion of adolescents who get adequate sleep (Healthy People 2030 and U.S. Department of Health and Human Services 2024). Sleep deprivation throughout adolescence is associated with both physical and mental health problems, including obesity, increased cardiovascular risk, diabetes, risky behaviors, and poor school performance (Shochat et al. 2014). Sleep difficulties often linger as adolescents progress into adulthood, accumulating sleep‐related consequences.

Across NDDs, it appears likely that sleep disturbances result from a combination of biological, psychosocial, environmental, and behavioral factors (Richdale and Schreck 2009). As sleep problems worsen and family tensions rise, parents become desperate for any intervention to provide relief (Waldron et al. 2016). The high prevalence of sleep problems in children with NDDs and the potential negative consequences of sleep disturbance make appropriate intervention an urgent priority for many families (Belli et al. 2022; Bruni et al. 2025; Vriend et al. 2011).

Melatonin is a neurohormone synthesized in the brain's pineal gland, contributing to the body's sleep‐wake cycle. Abnormalities in melatonin physiology have been consistently reported in children and adolescents with NDDs (Moore et al. 2017; Nováková et al. 2011; Rossignol and Frye 2014; Tordjman et al. 2013), but at least one study found similar endogenous melatonin production in children with ASD compared to typically developing peers (Goldman et al. 2014). Exogenous melatonin usage has demonstrated utility in increasing total sleep time and improving sleep latency in children and adolescents with NDDs, with minimal side effects reported (Bruni et al. 2015; Parvataneni et al. 2020; Rossignol and Frye 2011). However, significant variability in over‐the‐counter melatonin dosages has been reported (Grigg‐Damberger and Ianakieva 2017). A study analyzed the purity of thirty‐one melatonin supplements and found that eight melatonin supplements contained serotonin, a neurohormone with a similar molecular structure to melatonin (Erland and Saxena 2017). Melatonin concentration differed by −87% to +478% of the label claim. For instance, one label claimed to contain 1.5 mg of melatonin per tablet, but testing revealed that the chewable tablet contained approximately 8.6 mg of melatonin (Erland and Saxena 2017). Concerns about the dosage and purity of melatonin supplements raise questions related to melatonin's safety, tolerability, and efficacy. Additionally, the Centers for Disease Control and Prevention reported an increase in melatonin use in children and adolescents, along with a startling rise in the number of melatonin accidental ingestions, raising safety concerns about the accessibility of melatonin in the home (Lelak et al. 2022).

Parents have reported that they value the perceived naturalness of exogenous melatonin (Waldron et al. 2016) and may favor exogenous melatonin over other medications prescribed for sleep in their children (Lunsford‐Avery et al. 2016; Moore et al. 2017; Owens 2008). One survey study of parents of children with ASD found that 45% of parents reported exogenous melatonin use in their child, ranking melatonin as one of the most effective CAM therapies (Hopf et al. 2016). A study conducted in Australia, where melatonin is only available via prescription, used semi‐structured interviews to explore thirteen parents' experiences with exogenous melatonin usage among children 5–13 years of age with NDDs (Waldron et al. 2016). This study reported that all parent participants perceived melatonin to effectively address their child's sleep problems (Waldron et al. 2016). A study in the United Kingdom, another country where melatonin is only available via prescription, conducted focus groups with twenty‐six parents of autistic children and reported the value of the naturalness of melatonin (Horsnell et al. 2023). Although all parents would recommend melatonin (if all other options were exhausted), parents reported mixed efficacy in resolving their child's sleep problems (Horsnell et al. 2023). Another study from the United Kingdom used two surveys (clinician‐focused and parent‐focused) to assess ten parents' and twenty‐three clinicians' perspectives of exogenous melatonin usage to treat sleep disorders in children and adolescents (Banta 2008). Their research concluded that parents value the usefulness of melatonin, and clinicians recognize melatonin as an effective treatment for insomnia (Banta 2008). A 2024 study out of Ireland (melatonin is a prescription‐only medication) conducted semi‐structured interviews with twenty‐six parents of children with ADHD to investigate parents' experience and perceptions of sleep problems in their children, with only some of the parents endorsing a beneficial effect of melatonin on their child's sleep (Bondopadhyay et al. 2024).

Sleep changes significantly over the lifespan. Adolescence is a developmental period characterized by rapid physical and psychological changes, including puberty and endogenous circadian rhythms (Uccella et al. 2023b). To our knowledge, there have been minimal studies conducted in the United States that have focused on parents' experiences and perceptions of exogenous melatonin usage in adolescents with NDDs who report sleep problems. In most countries, exogenous melatonin is not available over‐the‐counter, requiring a prescription. Over‐the‐counter availability in the United States may lead to different patterns of exogenous melatonin usage and perceptions among parents. A thorough understanding of these perceptions and use patterns is important for pediatric clinicians in treating sleep disturbances in adolescents with NDDs. The purpose of this study is to describe parents' perceptions of exogenous melatonin usage in their older children (defined hereafter as adolescents 11–18 years of age) with NDDs.

## 2 Materials and Methods

Due to the exploratory nature of this study, a descriptive qualitative approach was utilized. The objective of this study was to describe parents' perceptions and experiences with exogenous melatonin usage in their child/adolescent with an NDD. To accomplish our objective, we aimed to (1) describe parents' perspectives of their child's past and current sleep problems, including the impact on family and trigger to seek treatment for sleep disturbance; (2) describe the rationale as to why parents chose exogenous melatonin to treat sleep problems or why exogenous melatonin was discontinued; (3) explore parents' perceptions of the proposed etiology, mechanism of action, and side effects of exogenous melatonin usage. Our university's Institutional Review Board approved this study (041‐18‐EX).

### 2.1 Participants

A convenience sample of twenty‐six parents of adolescents diagnosed with an NDD were recruited from a Midwest university medical center. Fliers were displayed in clinic offices, and researchers contacted potential participants during adolescent office visits. A Facebook advertisement was posted to the university's social media accounts. Potential participants interested in this study contacted the principal investigator via email and were screened for inclusion. A checklist of inclusion/exclusion criteria (Table 1) was utilized to identify participant eligibility. All participants who agreed to participate completed verbal consent for individual telephone interview sessions and agreed to an audio recording and transcription of the interview. Participants were compensated for their time.

**Table 1:** Inclusion & Exclusion Criteria.

Inclusion	Exclusion
Parent of an adolescent 11–18 years of age and consistently living with parental supervision	Parent report of adolescent with a known sleep disorder (e.g., sleep apnea)
Diagnostic report of confirmed NDD diagnosis by an appropriate provider or met DSM‐V criteria	Adolescent with NDD with a known genetic etiology (e.g., Angelman syndrome)
Previous use (within past 3 years) or current use of exogenous melatonin in their child	

### 2.2 Data Collection

**Figure 1:**
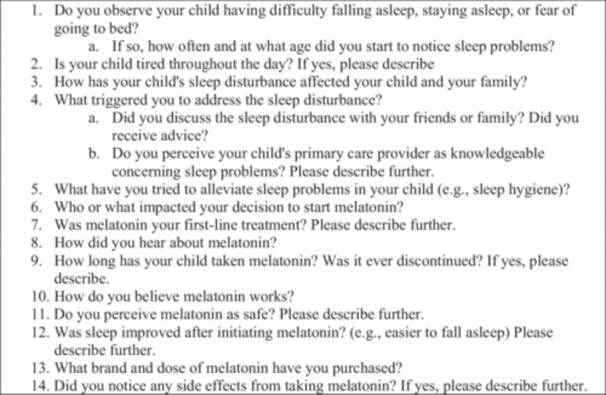
Interview guide.

Parent(s) were required to complete one interview. The primary investigator (AEH) conducted the interviews via telephone at a time convenient and private for participants. A semi‐structured interview guide (Figure 1) was utilized to guide the interview. Open‐ended questions were developed to promote deeper exploration of parents' perception of exogenous melatonin and stimulate further discussion. Questions were developed with clinical experts, research experts, and people with lived experience. Question topics included: (1) adolescent sleep disturbance, (2) influence to seek treatment, (3) perception of exogenous melatonin, (4) cultural dimensions influencing decision, (5) exogenous melatonin influence on sleep, and (6) involvement of adolescent's health care provider. A graduate assistant (AJK) transcribed the audio interview recording verbatim.

### 2.3 Data Analysis

Each interview was audio‐recorded, transcribed verbatim, and checked for accuracy. Conventional content analysis was employed (Hsieh and Shannon 2005). Two members of the research team individually coded each transcript (AH & SF). If a discrepancy in coding occurred, a third member of the research team (MS) resolved inconsistencies. The data was organized and examined to ensure that participants' identities were unknown. NVivo 14, qualitative data analysis software, was utilized for data management, organization, and thematic analysis. This software structured our data, allowing the discovery of connections and new insights, leading to an effective way to probe data questions. Data were collected until thematic data saturation (Berger et al. 2008).

## 3 Results

Twenty‐six individual interviews, lasting 20–30 min, were conducted with parents of a child with an NDD. The age of children diagnosed with an NDD ranged from 11 to 18 years. Of those children, seven were diagnosed with ASD, nine were diagnosed with ADHD, and ten were diagnosed with both ASD and ADHD. All parents completing an interview reported current melatonin use with their child or having used melatonin within the last 3 years with their child. The number of years parents had administered melatonin to their child ranged from 1 to 12 years. All parents reported that their child had trouble falling asleep before melatonin usage. The majority also reported difficulty staying asleep (*n* = 17). Most (*n* = 20) stated that their child's sleep difficulties were experienced nightly. Nearly half of parents (*n* = 12) reported first noticing sleep difficulties in their child between six and 9 years old. Some parents (*n* = 6) reported that their child had experienced sleep difficulties since birth. Five main themes emerged from individual interviews (Tables 2 and 3).

**Table 2:** Thematic Matrix.

Emergent theme	Theme cluster	Number of participants where theme was identified
Sleep disturbance and sleep‐related impairment	Difficulty falling asleep	26
	Negative child impacts	
	Tired	14
	Crabby/Irritable/Moody	11
	Hyperactivity/Symptom exacerbation	3
	Impaired concentration/functioning	2
	Negative family impacts	
	Sleep disruption	10
	Sleep deprivation	6
	Annoyances/Arguments/Frustrations	5
	Tired	2
	Decreased socialization/Strained relationships	2
Melatonin among the abundance of sleep interventions	Electronics restrictions	11
	Consistent nighttime routine	10
	Lavender/Essential oils	7
	Weighted blankets	3
Melatonin initiation	Provider recommendation	16
	Family/Friend recommendation	5
	Personal use/research	4
	Social media	1
Melatonin life	Decreased sleep latency	26
	Decreased nighttime awakening	12
Safety and naturalness	Melatonin is safe	24
	Melatonin is natural	15

**Table 3:** Illustrative quotes.

Theme	Illustrative quote
Sleep disturbance and sleep‐related impairment	“Without the melatonin he could take two to 3 h to go to sleep at night.” “Umm, he has a hard time going to sleep. He′s just awake.” “We′d go in there and he′d be reading and so you know we′d have to turn off the light and get him back to sleep. And some nights he would stay up until, on a school night, till 10 or 11 at night just because he said he couldn′t fall asleep.” “My brain just goes buzz buzz buzz and won′t stop.” “He wasn′t even 24‐h old, and he fought sleep.” “During the day, if he doesn′t sleep well at night, he gets kind of irritable and just out of it, not himself. He gets you know more angered easier.” “He is stressed about waking up because he doesn′t want to wake up, then he feels like he needs to get up and do something.” “He will kind of be slow to follow directions…he′s just not as tolerant of me giving him chores. He′ll do them but not as tolerable.” “I don′t sleep unless he/she does.” “I wouldn′t go to bed and go to sleep until I knew he/she was asleep.”
Melatonin among the abundance of sleep interventions	“…we tried other natural options like always using lavender uhh whether it be soap or umm lotions or oils. We tried things like that you know since he was a baby.” “We tried the behavioral stuff first.” “Like when he was really little, we made sure like we′d have the lavender bath you bath stuff… And to try to get him calmed down. We would give him a bath every night to you know try and wear him out like as a baby. You know and try and wear him out. Then umm, you know we′d try and do the same routine at bedtime. You know, doing the same thing reading a stories, getting get up and brushing his teeth. You know all that stuff. Umm, I know we tried some type of herbal type uhh medication first. Umm, before we went to uhh the melatonin.” “…we try to eliminate the liquids. We try to umm, we try to make sure he′s not doing screens right before he goes to bed. Umm so we′ll try to do either reading or playing a game or something you know an hour, hour and a half before it′s going to be bedtime so that he doesn′t have that extra stimulation.” “… in the basement because he′ll get up and go look at the clocks. Umm so we don′t have ANY clocks down in the basement umm for him to stress and worry about and we take his watch and any electronic devices out of his room at night so that he is not tempted to umm get up and look at those or play with those. Umm even if he has a particular book that he is obsessed with, we′ll take that out of the room at night.”
Melatonin initiation	“Our pediatrician recommended it a long time ago.” “I think it was just our recommendation by [insert provider name]. And then we were able to find it in the store and so we started using it.”
Melatonin life	“Once he fell asleep, he wasn′t having problems staying asleep.” “I think in general it helps overall.” “It probably takes her 20 min [to fall asleep] where it used to take her 45 min to an hour.”
Safety & naturalness	“Oh yeah, it′s free of chemicals,” “[Melatonin is] a little bit more natural than a specific sleep aid.” “Yeah, because I know it′s naturally found in, in your body system.”

### 3.1 Sleep Disturbance and Sleep‐Related Impairment

*“His brain can't turn off”*

Before the initiation of melatonin, all parents reported their child experienced difficulties falling asleep, with the majority reporting it as a nightly occurrence. Parents explained, “Without the melatonin, he could take two to 3 h to sleep at night.” One parent stated, “We'd go in there, and he'd be reading, and so you know we'd have to turn off the light and get him back to sleep. And some nights, he would stay up until, on a school night, till 10 or 11 at night just because he said he couldn't fall asleep.” Parents reported their child said their brain cannot turn off when trying to go to bed, or “My brain just goes buzz, buzz, buzz, and won't stop.” One parent described their child's inability to fall asleep as “He's just AWAKE!” One parent discussed how their child's difficulty falling asleep sets the tone for the week. “If I can't fall asleep tonight, then I'm going to be tired tomorrow, and I'm going to start the week bad.”

Parents reported their children exhibited a spectrum of negative emotional and behavioral impacts because of their child's sleep difficulty, which impacted their child's mood. Parents said, “During the day, if he doesn't sleep well at night, he gets kind of irritable and just out of it, not himself. He gets you know more angered easier.” More frequent emotional outbursts were reported, as well as increased child stress. Parents stated their child exhibited stress when going to sleep. “He is stressed about waking up because he doesn't want to wake up, then he feels like he needs to get up and do something.” Effects on cognition were also noted. “He will kind of be slow to follow directions… he's just not as tolerant of me giving him chores. He'll do them, but not as tolerable.” Many parents stated adverse effects on their child's academic performance, affecting their participation, schoolwork, and ability to plan for the day. Frequent reports of children falling asleep during class time were revealed.

Parents frequently reported negative family impacts of sleep difficulties in their children. A common sentiment among parents was “I don't sleep unless he/she does” or “I wouldn't go to bed and go to sleep until I knew he/she was asleep.” Frequent child nighttime awakenings often disturb parental sleep. Some parents reported that their child woke up several times throughout the night to go to the bathroom, eat a snack, or read because they could not fall back asleep. These sleep disturbances in the child/parent dyad also impacted the parent's ability to function while at work or while maintaining social roles. One parent described this by stating, “You have to get up and go to work and function, and that's very difficult to do on minimal sleep.” One parent also described these sleep disturbances as “just stressing.”

### 3.2 Melatonin Among the Abundance of Sleep Interventions

*“We tried the behavioral stuff first.”*

Insufficient sleep and sleep disturbances affected both the parent and the child, leading to a feeling of desperation. Parents reported utilizing a multitude of interventions to improve their child's sleep. One parent stated, “We tried the behavioral stuff first.” Another parent reported removing all clocks from the basement, saying, “He'll get up and look at the clock. So, we don't have ANY clocks down in the basement for him to stress and worry about.” Parents also reported using lavender‐based products, reading bedtime stories, and implementing sleep hygiene practices to decrease stimulation. Most parents did not use melatonin as a first‐line treatment to help their child sleep. Parents described this process of trial and error as discouraging. One parent described this as “just mom frustration.”

### 3.3 Melatonin Initiation

*“Some of them swear by melatonin.”*

Many parents reported discussing their child's sleep difficulties with their primary care providers. Most parents perceived their provider was knowledgeable about sleep disturbances, with some reporting their provider recommending melatonin supplementation. One parent stated, “Our pediatrician recommended it a long time ago.” Some parents also reported discussing their child's sleep difficulties with friends and families; however, parents perceived these discussions as not always helpful. When asked about discussing sleep difficulties with friends, one parent reported, “Some of them swear by melatonin.” One parent reported discussing their child's sleep difficulties with many different parties, “We discussed it not only with friends and family but with his pediatrician also.”

When asked about what prompted the use of melatonin, parents reported an exhaustion of resources and wanting to do what's best for their child. One parent stated, “You know, you only want the best for your kid.” Another parent reported that their decision to try melatonin was the result of “my lack of sleep and increased stress, as his as well.” One parent decided to give their child melatonin after discussing it with the child's provider: “It wasn't until [provider name] mentioned that it would be safe for us to try it on him.” Another parent described the final decision to try melatonin to “better his behavior during the day at school.” After reading about other parents' success in a social media group, one parent felt more comfortable trying melatonin. Several parents described their child's insufficient sleep as the trigger to try melatonin, “Well, just the fact that I saw that he couldn't get to sleep at night.”

### 3.4 Melatonin Life

*“I think, in general, it helps overall.”*

All parents reported that melatonin, in some way, improved sleep in their children. For example, all parents reported some decrease in sleep latency for their child, and about half of the parents mentioned decreased nighttime awakening. For example, “It probably takes her 20 min [to fall asleep] where it used to take her 45 min to an hour.” Minimal side effects were discussed and were not perceived as a barrier to melatonin usage. However, over half of the participants reported discontinuing melatonin during specific periods, with many taking breaks during the summer months. Regarding melatonin usage, participants stated they “Only use it during stressful periods where he is not sleeping well” or would “Take a break for 1 month – 6 weeks at a time.”

### 3.5 Safety and Naturalness

*“It's all‐natural”*

Most parents perceived melatonin as safe, and many described it as “natural.” One parent stated, “I know it's naturally found in, in your body system,” with another saying, “You know it's something that your body naturally produces.” When asked if he/she believed melatonin was safe, one parent stated, “Oh yeah, it's free of chemicals.” Compared to other sleep medications, one parent said, “[Melatonin is] a little bit more natural than a specific sleep aid.” One parent also perceived melatonin as non‐habit forming: “It's not like something you're going to become addicted to.” A few parents reported melatonin as safe in age‐appropriate doses or when being monitored by a health care provider.

When asked about the brand of melatonin used, many parents did not remember a specific brand, nor did they have brand loyalty. Parents stated, “It depends on where I remember to buy it” or “Whatever is cheapest.” When asked if parents noticed brand differences, some reported, “He seems fine regardless of the brand” or “I haven't paid attention to differences between brands.” One participant reported a new onset of nightmares with the use of a different melatonin brand. Several parents reported purchasing melatonin at a convenient place, such as a store they visited regularly. Most parents were knowledgeable about the dose of melatonin, but some parents were unsure of the dose and frequently reported switching dosages.

## 4 Discussion

To our knowledge, this is the first study conducted in the United States, where melatonin is available without prescription, focused on describing parental perceptions of and experiences with exogenous melatonin usage in children and adolescents with NDDs. The objective of this study was to describe parents' perceptions and experiences with exogenous melatonin usage in their older children and adolescents with NDDs. All participants reported sleep improvements in their children after initiating melatonin. Many parents reported positive differences in their child's ability to fall and stay asleep. Most parents reported that melatonin was not their first line of treatment when addressing their child's sleep difficulties. Parents reported the usage of many behavioral interventions before initiating melatonin, such as rigid bedtime routines, restriction of electronic devices, soothing music, weighted blankets, and baths or lotions with essential oils, such as lavender. Many of these behavioral interventions are congruent with the recommended sleep hygiene intervention as the first line of treatment (Blackmer and Feinstein 2016).

Most participants viewed melatonin as safe to administer to their children and perceived melatonin as natural. These findings were like those of Waldron et al. (2016), which were described as valuable to parents when considering interventions for sleep disturbances in young children with NDDs. It is important to note that the study by Waldron et al. (2016) was conducted in Australia, where melatonin remains available only by prescription. In the United States, melatonin is available as an over‐the‐counter nutritional supplement. Waldron et al. (2016) reported the perceived naturalness of melatonin, even though melatonin is a Schedule 4 prescription medication in Australia. The perception of melatonin as “natural” appears to be shared by parents whose children are prescribed melatonin (Waldron et al. 2016) and those whose children take over‐the‐counter melatonin with or without healthcare provider oversight.

Strengths of this study include a focus on over‐the‐counter melatonin (without prescription) from the perspectives of parents of a child with an NDD, including the rationale for starting melatonin, impact on the child and family, and real‐life usage (e.g., brands & dosages). Limitations of the study include only subjective parental perspectives and a lack of child or adolescent perspectives. Although cultural dimensions were included in the topic questions, no themes emerged. Future studies would benefit from validating and standardizing age‐appropriate adolescent sleep‐related interventions. Investigating the everyday usage and long‐term safety of exogenous melatonin is paramount for adolescents with NDDs and their families.

## 5 Conclusion

This study found that parents of adolescents with NDDs perceive melatonin to be a safe and effective intervention for managing their child's sleep‐wake disturbances. Parents expressed desperation for improved sleep health, as insufficient sleep among adolescents with NDDs can exacerbate negative behaviors and impact the family unit.

## How Might This Information Affect Nursing Practice?

Most parents reported switching between melatonin brands often based on convenience and cost. Given the reported inconsistencies in purity and dosage between brands, this is an important consideration for patient education and the potential impact of over‐the‐counter melatonin efficacy and safety.

## Conflicts of Interest

The authors declare no conflicts of interest.

## Supporting information

2024‐9‐16 (title page) supporting file not for review.

## Data Availability

The authors have nothing to report.
