# Supplementary material for: Desperate for Sleep: Exploring Parental Perceptions of Melatonin Use Among Adolescents With Neurodevelopmental Disorders
Source: J Spec Pediatr Nurs. 2025 Nov 1;30(4):e70010. doi: 10.1111/jspn.70010 (PMC12579445; doi:10.1111/jspn.70010)
Supplement: Supplementary file 1 — 2024‐9‐16 (title page) supporting file not for review. [file JSPN-30-e70010-s001.docx]

**Desperate for Sleep: Exploring Parental Perceptions of Melatonin Use Among Adolescents
with Neurodevelopmental Disorders**

Alyson E. Hanish, PhD, MSN, RN^1^, Shelby M. Freudenburg, DNP, APRN, RN^1^, Abbey J. Klein, PhD, FNP-BC^1^, Danielle J. Stappert, BSN, RN^1^, & Marcia Y. Shade, PhD, MSN, RN^1^

^1^University of Nebraska Medical Center, College of Nursing, Omaha, NE

*Corresponding author and person to whom reprint requests should be addressed:*

Alyson E. Hanish, PhD, MSN, RN

Assistant Professor, University of Nebraska Medical Center

985330 Nebraska Medical Center, Room 50110, Omaha, NE 68198 USA

Telephone: +1-402-559-3010

Fax: +1-402-559-8188

e-mail: [alyson.hanish@unmc.edu](mailto:alyson.hanish@unmc.edu)

The authors report no actual or potential conflicts of interests.

**Acknowledgement.** This research was funded by an internal research award through the University of Nebraska Medical Center, College of Nursing.
